# Patient and Surgeon Expectations Prior to Anterior Cruciate Ligament Reconstruction

**DOI:** 10.1007/s11420-018-9623-7

**Published:** 2018-08-13

**Authors:** Mahmoud Michael Khair, Hassan Ghomrawi, Sean Wilson, Robert G. Marx

**Affiliations:** Hospital for Special Surgery, 535 East 70th Street, New York, NY 10021 USA

**Keywords:** anterior cruciate ligament, expectations, ACL reconstruction surgery

## Abstract

**Background:**

When discussing potential treatment with patients choosing to undergo surgery for disruption of the anterior cruciate ligament (ACL) and their families, surgeons spend considerable time discussing expectations of the short- and long-term health of the knee. Most of the research examining patient expectations in orthopedic surgery has focused largely on arthroplasty.

**Questions/Purposes:**

The purpose of this study was to quantitatively assess the differences between the patient’s and the surgeon’s expectations before primary anterior cruciate ligament reconstruction (ACLR).

**Methods:**

In this case series, we prospectively enrolled 93 patients scheduled for primary ACLR between 2011 and 2014. Expectations were measured using the Hospital for Special Surgery 23-item Knee Expectations Survey; scores were calculated for each subject.

**Results:**

In all but six categories, patients had expectations that either aligned with their surgeons’ or were lower. The largest discordance between surgeon and patient expectations in which the patient had lower expectations was employment; 75% of patients had similar expectations to the surgeon when asked if the knee would be “back to the way it was before the problem started,” less than 1% had higher expectations, and 17% had lower expectations.

**Conclusion:**

In general, patient expectations align well with surgeon expectations. Patients who are older, have a lower activity level, and who have selected allograft over autograft for ACLR could also be at risk for greater discordance. Understanding these differences, and their predictors, will help guide physicians when they are counseling patients about ACLR and also help them interact with patients after surgery as they assess outcomes.

**Electronic supplementary material:**

The online version of this article (10.1007/s11420-018-9623-7) contains supplementary material, which is available to authorized users.

## Introduction

The study of patient expectations is becoming increasingly important as patient-based clinical outcomes have emerged as a large part of the evaluation of orthopedic interventions. While it is known that patient expectations are important, it is only recently that researchers have tried to measure them using valid and reliable instruments [1, 2, 6, 8, 13, 14, 18–21, 25]. Currently, most of the research examining patient expectations in orthopedic surgery has been in the arthroplasty literature [3, 4, 6–8, 11, 12, 15–17, 23–25]. This study was designed to explore patient expectations in one of largest subgroups of patients undergoing elective orthopedic surgery, those choosing to have anterior cruciate ligament reconstruction (ACLR).

Disruption of the ACL is one of the most frequent musculoskeletal injuries in active men and women. Recent estimates of the incidence of ACL injury are as high as 1:3000 per year, and there are approximately 175,000 ACLRs performed annually in the USA [5]. Some patients have surgery to eliminate symptoms of instability, while others do so soon after injury to prevent future instability.

When caring for these patients and discussing potential treatment options, surgeons spend a considerable amount of time with the patient and their families discussing expectations with regard to the short-term and long-term health of the patient’s knee. This is further complicated when the patient is young and has ambitions to play demanding sports at a high level. Despite the efforts of the surgeons and staff to educate patients, the extent to which the surgeon’s expectations of ACLR align with a patient’s remains unknown. The aim of this study was to quantitatively assess the differences between patient and surgeon expectations prior to primary ACLR. Understanding these differences will help guide surgeons when they are counseling patients about ACLR and when interacting with patients after surgery.

## Materials and Methods

After obtaining written informed consent, we prospectively enrolled 93 patients scheduled for primary ACLR between 2011 and 2014 from the practice of the senior surgeon (RGM). Patients were asked to enroll if they were 14 years of age or older; had a mechanism of injury, clinical examination, and magnetic resonance imaging (MRI) scan supporting a diagnosis of ACL rupture; and were indicated for primary ACLR. Patients who had a history of surgery on either knee, who refused to be in the study, or who had a history of psychological illness that prohibited them from filling out the necessary forms were excluded. Patients were enrolled by one surgeon when they were identified as meeting the inclusion criteria. Females made up 38.7% of the cohort and males 61.3%. The mean age of the patients in the cohort was 27.9 years (range, 14 to 60 years). None of the patients were receiving worker’s compensation benefits.

Expectations were measured using the Hospital for Special Surgery (HSS) 23-item Knee Expectations Survey [13]. The survey has been shown to have face, content, and construct validity, as well as high construct and test-retest reliability [13]. It has a patient format and a surgeon format that contains identical items and was developed to evaluate expectations of recovery from ACLR. Measures include pain relief, improved knee mobility, knee stability, and ability to exercise and participate in sports. The improvement expected on each item was measured using a 4-point Likert-type scale (1, back to normal or complete improvement; 2, not back to normal but a lot of improvement; 3, not back to normal but a moderate amount of improvement; 4, not back to normal but a little improvement). Each item also had a no-expectation option (5, I do not have this expectation, or this expectation does not apply to me).

After patients were enrolled and the surgeon discussed the risks and benefits of surgery, the patient and the surgeon each filled out the expectations survey and were blinded to the other’s responses. In addition, each patient was asked to provide baseline demographic data and complete a Marx Activity Level Scale [22] and International Knee Documentation Committee (IKDC) survey [9, 10].

The Marx Activity Level Scale is a discriminative instrument that consists of four questions and takes patients about a minute to complete. It was designed to capture functional activity rather than sports-specific activity. Specifically, it assesses running, cutting, pivoting, and decelerating. The items are scored 1 to 4, depending on the frequency the activity is performed per month. The scale is easy and quick to use, is a validated instrument, and captures important information on patient function [22].

The IKDC survey is a joint-specific rather than a condition- or disease-specific instrument. It evaluates symptoms, function, and sports activity for a number of different knee conditions. The subjective form contains 18 questions. The raw score can in turn be transformed into a score from 0 to 100, where 0 is the worst possible score and 100 is the highest. The IKDC has been validated and has also been shown to be responsive and reliable for a variety of knee conditions, including ACL tears [9, 10].

Patient and surgeon expectation scores were calculated for each subject by adding the scores for all of the questions and converting the raw data to a 0-to-100 scale, with 100 being the highest expectation of returning to a baseline normal state in all ways and 0 being an expectation of no improvement at all in any category. Items for which the respondent had no expectation were given no weight in calculating the score.

Descriptive statistics were performed using means and standard deviations for continuous variables and frequencies for binary and categorical variables. Discrepancy between patient and surgeon expectations was assessed at the item level and at the cumulative score level. At the item score level, non-parametric Wilcoxon signed-rank test was performed to determine agreement between patient and surgeon responses to the same item. At the cumulative score level, the difference between patient and surgeon cumulative scores was calculated and then regression analysis performed to determine predictors of disagreement in cumulative expectation scores. All analyses were conducted in SPSS 18.0 (IBM/SPSS Inc., Armonk, NY, USA). The institutional review board at our institution approved this study.

## Results

In all but six categories, patients had expectations that either aligned with the surgeon’s or were lower (Table 1). The largest discordance between surgeon and patient expectations in which the patient had lower expectations was in employment. Only 60% of the time did the patient agree with the surgeon on the effect of surgery on the patient’s future employment; 35% of patients had lower expectations than the surgeon and none had higher expectations. Another large discordance was in the expectation of being able to participate in professional sports: 59% of patients had higher expectations in this category than the treating surgeon, 41% had the same, and no patient had lower expectations. Perhaps most important, 75% of patients had similar expectations to the surgeon when asked if the knee would be “back to the way it was before the problem started.” Less than 1% had higher expectations and 17% had lower expectations.

**Table 1:** Patient expectations compared to surgeon expectations

Expectation	Patient expectations compared to surgeon expectations			*κ* (*p* value)
	Higher	Same	Lower	
Pain relief ^a^	0	80	13	0.4 (< 0.001)
Walking short distance ^b^	0	86	7	0.3 (< 0.001)
Walking medium distance	0	85	8	0.3 (< 0.001)
Walking long distance ^b^	0	81	12	0.2 (< 0.001)
Increasing in knee stability	0	78	15	0.2 (0.003)
Increasing in knee mobility ^c^	0	78	15	0.2 (0.001)
Going up and down stairs, (one missing)	0	86	6	0.4 (< 0.001)
Squatting ^c^	0	73	20	0.2 (0.002)
Kneeling ^c^	11	67	15	0.2 (0.009)
Stopping knee from catching or buckling ^d^	0	78	15	0.18 (< 0.001)
Stopping knee from giving way ^b^	0	79	14	0.2 (< 0.001)
Stopping knee stiffness	1	77	15	0.3 (< 0.001)
Employment ^e^	0	56	32	0.1 (< 0.001)
Running	0	86	7	0.4 (< 0.001)
Performing daily activities	0	86	7	0.3 (< 0.001)
Exercising	2	82	9	0.2 (0.005)
Participating in professional sports ^f^ (one missing)	55	37	0	0.1 (0.001)
Having confidence in the knee	0	82	11	0.2 (< 0.001)
Avoiding future knee degeneration	10	69	14	0.2 (0.008)
Maintaining general health	0	84	9	0.3 (< 0.001)
Interacting with others	0	84	9	0.3 (< 0.001)
Psychological well-being ^g^	0	77	16	0.2 (< 0.001)
Knee back to the way it was before the problem started	7	70	16	0.1 (0.201)

^a^2 patients do not have this expectation

^b^3 patients do not have this expectation

^c^1 patient does not have this expectation

^d^4 patients do not have this expectation

^e^27 patients do not have this expectation; the surgeon does not have this expectation for 1 patient

^f^25 patients do not have this expectation; the surgeon does not have this expectation for 80 patients

^g^10 patients do not have this expectation

Overall, surgeons had a mean cumulative expectation score of 94.9 (range, 75 to 100), while patients had a mean cumulative score of 91.3 (range, 47.8 to 100). The mean patient expectations score was significantly lower than that of the surgeon (difference = 3.98; *p* < 0.01) with a wide range of differences (− 45.0 to 7.6). However, 68 patients had a cumulative score within 5 points of their surgeons’ scores. When regression analysis was conducted, the difference in expectations scores between patients and surgeons was 15.1 points (CI, − 27.2 to − 3.1; *p* ≤ 0.014), smaller in patients undergoing allograft than in those undergoing autograft reconstruction. Similarly, the difference was smaller with age (difference decreases by 0.5 points [CI, − 1.0 to 0; *p* ≤ 0.052] for every year increase in age). Finally, as the pre-operative Marx Activity Level Scale score increases, the discordance in scores increases (difference increases by 0.5 points [CI, 0 to 1.1; *p* < 0.057] for every unit increase in activity score). All other predictors examined did not prove to be significant.

## Discussion

The results of this study showed that in the senior author’s clinic, a majority of the time, the surgeon and patient had similar expectations of the outcome of primary ACLR. In 17 categories, if the patient and surgeon did not have concordant expectations, the patient had lower expectations. In the other six categories, patients had higher expectations a minority of the time, with the exception of “participating in professional sports,” where 59% of patients had higher expectations than the surgeon and none had lower expectations. The greatest discordance in which the patient had lower expectations than the surgeon was in predicting the effect of primary ACLR on the patient’s future employment. Significant predictors of discordance between the surgeon and the patient include patients who are older, those with lower pre-operative Marx Activity Level Scale scores, and those undergoing allograft instead of autograft reconstruction.

There are several limitations to this study. The first is that it represents a single point in time in the patient–physician dialog. Future studies would benefit from long-term follow-up data to examine whether and how patient and surgeon expectations change as patients recover from primary ACLR. While this study is applicable and reflective of the initial expectations that both surgeon and patient have at the time of surgical indication, it is unknown how these expectations evolve over time. Second, this study was performed in one surgeon’s clinic and may not be generalizable to other settings.

There is one other study in the literature to date that examines patient expectations of primary ACLR. Feucht et al. looked at patient expectations in both primary and revision ACLR using a non-validated five-item questionnaire that the authors developed [2]. They prospectively collected data on 181 consecutive patients undergoing either primary ACLR (73%) or revision ACLR (27%) and had enrolled patients fill out a questionnaire 24 to 48 h before surgery. They found that all patients had the expectation that after surgery the affected knee would be normal (38%) or nearly normal (62%). Most patients expected to return to sport at the same level (67%) and also experience no instability independent of activity (77%). Slightly more than half of the patients (58%) expected no pain independent of activity level, and half (50%) expected no increased risk of osteoarthritis. As expected, the revision group had significantly lower expectations than the primary ACLR group on overall condition, pain, and return to sport. That study did not evaluate surgeon expectations; therefore, those authors were unable to correlate surgeon and patient expectations, as we did.

In conclusion, patient expectations on ACLR align well with surgeon expectations most of the time. Future employment and return to professional sport are two areas where the surgeon and patient have significant discordance. Patients who are older, have a lower activity level, and have selected allograft over autograft for ACLR could also be at risk for greater discordance.

## Electronic supplementary material

ESM 1 (PDF 1224 kb)

ESM 2 (PDF 1225 kb)

ESM 3 (PDF 1224 kb)

ESM 4 (PDF 1224 kb)
